# Can formalizing links among community health workers, accredited drug dispensing outlet dispensers, and health facility staff increase their collaboration to improve prompt access to maternal and child care? A qualitative study in Tanzania

**DOI:** 10.1186/s12913-017-2382-1

**Published:** 2017-06-19

**Authors:** Angel Dillip, Suleiman Kimatta, Martha Embrey, John C Chalker, Richard Valimba, Mariam Malliwah, John Meena, Rachel Lieber, Keith Johnson

**Affiliations:** 1 Ifakara Health Institute, P.O. Box 78 373, Dar es Salaam, Tanzania; 2 Pharmaceuticals & Health Technologies Group, Management Sciences for Health, P.O. Box 50104, Dar es Salaam, Tanzania; 3 Pharmaceuticals & Health Technologies Group, Management Science for Health, 4301 North Fairfax Drive, Arlington, VA 22203 USA; 4 Kibaha District Council, P.O. Box 30153, Pwani, Tanzania; 5 Reproductive and Child Health Unit, Ministry of Health, Community Development, Gender, Elderly and Children, P.O. Box 3448, Dar es Salaam, Tanzania

**Keywords:** Maternal health, Newborn health, Child health, Community health workers, Referral, ADDO, Health facility

## Abstract

**Background:**

In Tanzania, progress toward achieving the 2015 Millennium Development Goals for maternal and newborn health was slow. An intervention brought together community health workers, health facility staff, and accredited drug dispensing outlet (ADDO) dispensers to improve maternal and newborn health through a mechanism of collaboration and referral. This study explored barriers, successes, and promising approaches to increasing timely access to care by linking the three levels of health care provision.

**Methods:**

The study was conducted in the Kibaha district, where we applied qualitative approaches with in-depth interviews and focus group discussions. In-depth interview participants included retail drug shop dispensers (36), community health workers (45), and health facility staff members (15). We conducted one focus group discussion with district officials and four with mothers of newborns and children under 5 years old.

**Results:**

Relationships among the three levels of care improved after the linkage intervention, especially for ADDO dispensers and health facility staff who previously had no formal communication pathway. The study participants perceptions of success included improved knowledge of case management and relationships among the three levels of care, more timely access to care, increased numbers of patients/customers, more meetings between community health workers and health facility staff, and a decrease in child and maternal mortality. Reported challenges included stock-outs of medicines at the health facility, participating ADDO dispensers who left to work in other regions, documentation of referrals, and lack of treatment available at health facilities on the weekend. The primary issue that threatens the sustainability of the intervention is that local council health management team members, who are responsible for facilitating the linkage, had not made any supervision visits and were therefore unaware of how the program was running.

**Conclusion:**

The study highlights the benefits of approaches that link different levels of care providers to improve access to maternal and child health care. To strengthen this collaboration further, health campaign platforms should include retail drug dispensers as a type of community health care provider. To increase linkage sustainability, the council health management team needs to develop feasible supervision plans.

## Background

The global burden of maternal, newborn, and child mortality has decreased considerably in recent years [1, 2]; the pace, however, is not the same from country to country. Most developing countries, including many in Africa, were far from achieving the Millennium Development Goals 4 and 5 for maternal, newborn, and child health by 2015 [3]. While Tanzania met its under-five mortality goal in 2012, progress toward decreasing maternal and newborn mortality was not as successful [4, 5]. The newborn mortality ratio, for example, was 21 per 1000 live births, and the maternal mortality ratio was 410 deaths per 100,000 live births compared with goals of 19 and 223, respectively [4, 6]. Maternal mortality in Tanzania has been associated with pregnancy and childbirth complications, such as hemorrhage, hypertension, and delay in obstetric care [4]. Among newborns, the primary contributors to mortality are prematurity, birth asphyxia, pneumonia, and delay in treating infections, while child deaths are mostly caused by pneumonia, diarrhea, and malaria—diseases that are easily surmounted if treated promptly [4].

Improving maternal, newborn, and child health is a priority in Tanzania’s *National Strategy for Growth and Reduction of Poverty II* [7]. In response to the country’s lagging performance related to decreasing newborn and maternal mortality, the President of Tanzania launched the Sharpened One Plan and Big Results Now initiatives in 2014 to use innovative approaches to accelerate the reduction of these deaths. Within this context, the Kibaha District Council, with support from Management Sciences for Health, responded by taking the existing structure of health services and providers and linking accredited drug seller outlet (ADDO) dispensers with local community health workers (CHWs) and health facilities (HFs) to improve community-based access to medicines and health care, particularly for women and children.

Many people in low-income countries access their medicines or health care from retail drug sellers [8, 9]. In Tanzania, a public-private partnership launched in 2003 used an accreditation approach to improve access to quality medicines and pharmaceutical services in these shops that serve primarily rural and peri-urban areas that are otherwise underserved. The ADDO program covers the nation, with over 11,000 shops now accredited. On the other hand, CHWs in Tanzania do not carry medicines, so their practice is to advise sick people to get medicines from a health facility, which may be some distance away. As part of this intervention, CHWs were officially empowered to refer cases in the community to ADDOs to receive a timely first dose of an appropriate medicine, including antibiotics, before arriving at the health facility. ADDO dispensers were already equipped with tools such as patient registers and referral forms, and they send reports via mobile phone every month to the Pharmacy Council, which is the regulatory oversight body; however, they do not have a formal linkage with health facilities. Likewise, CHWs have registers of sick people they encounter, referral forms, and reports that they submit to nearby health facilities on a monthly basis. The roles of CHWs and ADDOs in improving access to care have been widely documented [10, 11].

In June 2015, we organized a training in Kibaha district to create awareness among three categories of community-level health care providers and to highlight the potential of having them work together to improve prompt acess to maternal and newborn health. The health service structure in Tanzania comprises six levels: village health services provided by CHWs is the lowest level of health care delivery, the dispensary is a step up from that, while a health center is expected to serve a catchment area of 50,000 people. The next highest levels are district hospitals, regional hospitals, and referral/consultant hospitals. The training brought together 85 CHWs, 17 health facility staff from four levels of health care in the district—dispensary, health center, and district and referral hospitals—and 40 ADDO dispensers. The training focused on recognition of danger signs during pregnancy, after delivery, and in sick newborns; family planning methods; roles and responsibilities of CHWs, ADDO dispensers, and HF supervisors; the health care referral system at community level; and monitoring and supportive supervision. As the district’s linkage supervisors, Kibaha’s council health management team (CHMT) members and other district officials also received sensitization on the plan. We conducted two rounds of supervision in July and September 2015, and the results showed that between the two visits, trainees had increased their knowledge of danger signs and family planning methods, and health facility staff had attended more births.

Several studies have looked at how well integrating CHWs into health facility activities improves prompt access to care [12]; however, information is lacking on how private and public facilities could similarly collaborate. This study explored barriers, successes, and promising approaches to increasing timely treatment by linking three levels of care—CHWs, ADDOs, and health facilities. We hoped to use our findings to enhance interventions to strengthen the linkage and help reduce maternal and newborn deaths in Kibaha.

## Methods

### Study area

The study was conducted in Kibaha district in the Coast region of Tanzania in November 2015. According to the 2012 national census, the district has an estimated population of 198,697 inhabitants. The main economic activities include agriculture, livestock keeping, and small businesses.

### Study design and participant selection

We used a qualitative approach rooted in the principles of grounded theory [13], in which we continued sampling until we reached response saturation. We carried out in-depth interviews with ADDO dispensers, CHWs, and health facility staff. The study participants were purposively sampled to ensure that we obtained adequate information to achieve the study objectives and capture differences in responses among the three groups. In addition, we conducted separate focus group discussions with district officials and mothers of newborns and children under 5 years to gather the opinions of both the supervisors and beneficiaries of health care services. Our interview methods took an inductive approach that allowed participants to report issues related to the experience of working with each other, while we probed for necessary information related to our study objectives [14].

Our sample size was determined using saturation sampling and related to the number of participants who had received training [15]. The original training included 40 ADDO dispensers; however, for this study we interviewed 36, while using the other four to pilot test the data collection tool. The number of CHWs who received training was 85, and based on saturation principles, we aimed to include half of those trained in each ward [15]; in total, we interviewed 45 of them. At health facility level, 15 staff members from all four health care levels were included, with the exception of two who were involved in the pilot exercise. We conducted one focus group discussion with district health officials and four with mothers of newborns and children under five. We based our selection of district officials, who were CHMT members, on their role in supervising the linkage. The group included the district medical officer, district health secretary, reproductive and child health coordinator, district coordinator for chws, acting district pharmacist, acting district dental officer, district family planning coordinator, and district coordinator for neglected tropical diseases.

### Data collection procedures

We recruited and trained four experienced research assistants, two girls and two boys. We then piloted the data collection tools in Kibaha with four ADDO dispensers, four CHWs, and two health facility staff members. The in-depth interview guide was revised based on the results of the pilot for use in the actual data collection exercise.

The research assistants interviewed two groups of respondents at their workplaces (ADDOs and health facilities). The interview process was flexible enough to allow customers to access services between questions. Community health workers were interviewed at the health facilities where they receive supervision. Focus group discussions with mothers of newborns and children under five also took place at the health facility. The nursing officer in charge selected mothers of healthy newborns who had been in-patients, but who were about to be discharged. Mothers of small children were selected from those who had brought their children in for clinic services. The number of focus group participants ranged from 8 to 12 per group. Each indepth interview lasted for not more than an hour, while focus group discussion ranged between one to 1 hour and a half.

All interviews and focus group discussions were conducted in Kiswahili language. The senior social scientist checked the quality of the in-depth interviews by revisiting and interviewing some of the ADDO dispensers, CHWs, and health facility staff. All focus group discussions were facilitated by the senior social scientist, who is the first author, with assistance from a research assistant. We used recording devices; however, the research assistant also took notes, which were then expanded later [16]. All recorded interviews were transcribed.

### Data analysis and reporting

Data was analyzed using NVIVO 9 software [17]. Two persons conducted an inductive thematic analysis of transcripts to come up with codes, and we checked for coding consistency. Additional codes identified through the line-by-line coding were added. We reviewed the list of codes and grouped them into categories and themes for analysis. We analyzed them by comparing themes that related to our study objectives. We did not translate the data in advance; all data were analyzed in Kiswahili.

### Ethical considerations

We obtained ethical clearance from the National Institute of Medical Research of Tanzania. An information sheet about the study was drawn up in Kiswahili, explaining why the study was being carried out, by whom, and what it would involve. We assured respondents’ confidentiality. Respondents were asked if they had any questions and whether they agreed to take part in the study. We got the written consent of all respondents. We discussed the study in advance with the responsible district authorities to ensure their support.

## Results

The results are presented in the following themes: 1) relationships among CHWs, ADDO dispensers, and HF staff; 2) patient identification and referral and documentation; 3) perceived linkage successes; and 4) the linkage’s challenges and promising approaches. We grouped the findings from our interviews with CHWs, ADDOs, and HFs together and present the results of the focus group discussions with mothers and district health officials separately.

### Relationships among CHWs, ADDO dispensers, and health facility staff members

To learn more on how the linkage functions, we asked participants about their relationships with each other before and after the CHW–ADDO–HF training.

#### CHW–ADDO relationships

CHWs reported better communication with ADDO dispensers after the training. They felt that because the two groups had not worked together previously, their relationships had been informal at best, and some did not know each other personally. CHWs had previously been unhappy that dispensers perceived them as “uneducated” and perceived their cadre as lacking importance in the health field, “*The training has actually blended us, we now know each other. Of course before the training we used to greet each other as people who belong to the same community, but speaking frankly, dispensers did not value our contribution as care providers”* (CHW, Kibaha). CHWs appreciated the fact that they can now discuss health matters with dispensers who now regard them as fellow health care professionals. One reported, *“Nowadays you can pass at the outlet and friendly discuss with dispensers about patients’ complications; they listen to you, we now acknowledge each other’s contribution in health service provision. Even when you send a patient to the ADDO, he or she is well received. Before, they regarded us as people who did not go to school*” (CHW, Kibaha). Findings also revealed that because of the improved relationship between the two groups, ADDO dispensers provide some CHW-referred patients medicines on a loan basis with the CHW covering repayment if needed. A CHW said, “*Sometime we bail them* [patients] *out. They receive medicine on loan basis. At times, not all can afford to buy medicine, and once they pay, the dispenser informs us*” (CHW, Kibaha).

The same was true for ADDO dispensers who reported better relationships and communication with CHWs after the linkage training. Dispensers felt that in the past, CHWs had regarded them as business people and profit makers and not as care providers. This dispenser noted, “*Before the training, I did not know much about CHWs. I did not know what they were doing. At least I have come to understand their role*, *and after the training, we have become very close. I can take care of a patient for some time, and when I feel like she can continue with care at community level, I call the respective CHW to come and take his patient*” (dispenser, Kibaha).

#### CHW–HF relationships

When asked about their previous relationships, CHWs and HF staff reported having had an existing working relationship before the CHW–ADDO–HF training. However, the training reportedly cemented the relationships and increased the respect between the two levels. CHWs acknowledged that after attending the training. They received forms to refer patients to heath facilities, which made them feel part of the health profession team, “*Yes, we were close to each other, but after training, our relationship is now 200 percent. We are more serious now and respect each other’s role*” (CHW, Kibaha). A health facility staff member said, “*We have been working with CHWs, particularly on home-based care for people living with HIV. Of course, we have now become very close because they can come here any time, ask questions, and discuss challenges involved in identifying patients in the community*” (HF staff person, kibaha).

Following the intervention, CHWs and HF staff are supposed to meet once a month for CHWs to present their monthly reports. We noted however that CHWs who live close to the HF report at the facilities weekly or even daily because they regard the HF as their workplace, and they have been working with health facility staff on a number of other interventions, such as home-based care. The topics discussed most often during CHW–HF meetings are challenges in identifying danger signs among pregnant women, newborns, and children under five; women utilizing family planning services; and how CHWs can reach more patients to refer to either HFs or ADDOs.

#### ADDO–HF relationships

The relationships between ADDO dispensers and HF staff did not exist before the training, so post-training changes were more dramatic, as this health care provider noted, “*We did not have contact. We used to regard them as medicine sellers, but after training, we have become one. You can pass at the outlet anytime and discuss patient cases*” (HF staff person, Kibaha).

ADDO dispensers also felt strongly that the relationships had been strengthened, “*We used to regard each other as two separate people. Health facility staff used to value themselves as people who are more professional than us as business people who did not go to school. Patients who used to attend here before going to facilities were insulted by health care providers as to why they came to us first. They were told that they should go straight to the health facility. Honestly, after the training, things have very much changed, we are very close. A patient receives care at the community level through CHWs who advise her whether to come here or to go to the HF, depending on her health condition*” (dispenser, Kibaha). Another said, “*We now know each other quite well. During the training, we exchanged mobile phone numbers. Sometimes I receive seriously sick patients, and what I do is I refer them to the facility and call the respective staff in charge so that they attend my patient in time*” (dispenser, Kibaha).

### Referrals: Identification of patients, documentation, and feedback

CHWs reported identifying patients in need through household visits, reports from fellow community members, and consultations with patients themselves. Identifying patients in the community comes with challenges, the first being that CHWs are known in the community to support people living with HIV. For example, following up pregnant women who are living with HIV and who have not exposed their status to their husbands or other relatives is difficult because the women do not want to have contact with CHWs in case their status is revealed. This makes it hard for CHWs to advise them about their other health matters, “*I asked for her clinic card, and it is when I learned she is living with HIV. I asked if she is taking good care of her health as recommended, and she told me that I should reduce my voice because no one knows her status, and that I should not visit her frequently*” (CHW, Kibaha). Another challenge was that some patients prefer traditional to modern medicine; therefore, they refuse the CHWs’ advice to go to either the ADDO or HF, especially when they do not see quick improvement in their condition.

On average, in the month before the data collection, 33 referrals were documented from CHWs to health facilities, five from CHWs to ADDOs, and 22 from ADDOs to HFs. HF staff referred patients to ADDOs only when they had medicine stock-outs. To gain a deeper insight into why referrals from CHWs to ADDOs were few compared to those directed to HFs, some CHWs reported not having referral forms to refer patients to ADDOs, while others felt like referrals to HFs were more important because patients will undergo a different diagnostic procedure.

While some CHWs provided oral referrals to ADDOs, they rarely documented them. The same applied to ADDO dispensers who did not document referrals received from CHWs. When asked why, an ADDO dispenser said, “*I do not document patients referred to me by CHWs, this is because their referrals are oral, they do not come with any referral forms,*” and a CHW noted, “*We were not told to record patients we refer to ADDOs, and am not very clear about how to do that*” (CHW, Kibaha).

Probing more into the lack of referral forms and reasons for not providing oral referrals to ADDOs instead, we found that CHWs placed a high value on using referral forms, and they did not consider oral referrals as a professional practice. More importantly, the majority of CHWs felt that their decision of where to refer a patient was determined by his or her condition. The conditions that prompted referrals to ADDOs involved family planning services, especially medicine-related, and minor health complications among children under 5 years, such as headaches and stomach aches. ADDO dispensers also confirmed this. Most CHW referrals to the health facility were for conditions such as umbilical cord and eye problems and breathing complications among newborns; bleeding complications, leg swelling, constipation, and stomach ache among pregnant women; malaria and convulsions and diarrhea among children under five; leg swelling among women who have delivered; and women in need of family planning services, especially injections.

ADDO dispensers and CHWs were trained to document referrals to HFs in a special referral form. However, some CHWs and ADDO dispensers said they provided referrals using a piece of paper or did not provide anything at all. HF staff agreed, saying they did not always receive referral forms from CHWs or ADDOs and that sometimes CHWs use their home-based care books improperly to compile reports for other health conditions experienced by women and children. For example, one CHW said, “*Almost one year now and I do not have a referral book; sometimes I just write the referral on a piece of paper. We requested the book from the health facility, but we were told to use HIV referral books. I cannot use it because that is special for people living with HIV*” (CHW, Kibaha). ADDO dispensers had similar problems, “*It is has been some time now since my referral forms were finished. They promised to bring the forms…nothing has been done. Whenever I want to refer a patient, I just call the doctor direct and tell him such-and-such patient is coming*” (dispenser, Kibaha).

Some HF staff said they did not document referrals from ADDOs because it is the ADDO dispensers’ duty to report that information to the Pharmacy Council. Other staff felt that referrals from ADDOs are documented, but were not sure how it is done or who does it. Asking how they document referrals from CHWs, participants were not certain about the process, “*You know the referrals go directly to the doctor. I am sure he records them somewhere, although after the training, we told them that they should record it. I don’t know, but I think they do not*” (HF staff person, Kibaha).

With regard to feedback, HFs provided feedback to CHWs when a referral form had been used, because the referred patients are directed to bring a piece of paper back to the respective CHWs after visiting the health facility. In cases without a form, no feedback is provided, except from some patients who are followed up by CHWs themselves to find out what happened during their HF visits. The majority of ADDO dispensers said they got feedback from patients when they came in with prescriptions from health care providers, for example, “*In most cases, patients are advised to purchase medicines from the outlets, so when they come with the prescriptions they also tell us how it went at the health facility”*(dispenser, Kibaha).

### Perceived linkage’s successes

Participants expressed their views on what they saw as achievements in health care provision and collaboration as a result of the training.

#### Improved knowledge of case management

Participants, particularly CHWs, felt that after the training, they were more professional in how they handled different cases in the community, “*I did not know that women who have given birth also need to continue visiting health facility for some time, but I have come to know that, and we advise women that after birth, they should continue attending health facilities for further check-ups*” (CHW, Kibaha). An ADDO dispenser added, “*For example, now a child can come with diarrhea, and before referring her to the facility, I provide her with ORS so that she doesn’t lose water*” (dispenser, Kibaha).

#### Improved relationships with each other

Participants acknowledged their closer relationships since the training, and that everyone understands the role of his or her counterpart. They can have friendly discussions on how to deal with certain health conditions. This CHW emphasized, “*The training has widened us. We regard ourselves as one, and I clearly understand what is the role of dispenser and health care provider. A dispenser can call me and tell me about the health condition of a certain patient, and we discuss the way forward on how I should follow him up for further advice.*” While an ADDO dispenser added, “*In the past when you see a doctor from the health facility, you close your outlet, worrying that he might ask you many questions. Now we welcome them, they are our friends*” (CHW, Kibaha).

#### Prompter access to care

Participants thought that timely access to care has increased, especially among children under 5 years of age, due to their improved knowledge of danger signs. A health care provider said, *“Now things have improved. In the past, we used to receive children who were seriously sick. They were not arriving at the health facility in time; at least CHWs now send children at the right time*” (HF staff member, Kibaha). Related to this, CHWs reported that the health facilities had improved their services, “*Once you refer a child, for example, when he reaches the health facility, he immediately receives treatment, and in case there are no medicines, he is immediately referred to ADDO*” (CHW, Kibaha). Additionally, most of the CHWs felt that adherence to childhood vaccination protocols had improved compared to the previous year or two.

Participants also though pregnant women were getting better care. *“In the past, some women did not attend antenatal clinic until the belly becomes very big, but now that problem is over”* (CHW, Kibaha). Dispensers reported the same, *“Patients now receive timely treatment because they know where to start. When they visit the outlets depending on their condition, I direct them to which section to start with at the health facility. In the past, you could find a pregnant woman taking traditional medicines”* (dispenser, Kibaha). Moreover, CHWs and ADDO dispensers reported that they have now succeeded in reaching and educating pregnant women in pastoral communities. This was done through village health day programs that CHWs attend and by having ADDOs participate in remote areas. Such women have difficulty attending antenatal care because they travel to remote areas while taking care of their cattle.

#### Perceived decrease in number of maternal and newborn deaths

There was a perception among all CHWs that the number of deaths in the community had decreased, especially among newborns and pregnant women, compared to 1 or 2 years before as a result of women not attending antenatal clinics and delivering at home, “*Since the training, we have lost only one newborn in our village, compared to before where such incidences were high*” (CHW, Kibaha). CHWs had also initiated a strategy where during meetings with pregnant women, they told them that if they delivered at home rather than in a facility, their husbands or relatives would have to pay a fine. The CHWs felt that this had increased the number of facility-based deliveries. In addition, HF staff thought that because CHWs and ADDO dispensers were better able to identify danger signs among pregnant women and send them to the HF, maternal mortality had decreased.

#### More recognition from the community

Participants are being consulted more often by community members in need of health advice, as an ADDO dispenser said*, “Of course we like profit, but the training highlighted the side effects of medicines, and so for not all conditions do we sell medicines; sometimes patients need only health advice”* (dispenser, Kibaha). CHWs reported that they are now recognized by the village government and given a time slot to educate the community on health matters during village meetings, *“Now the community recognizes us because in the past we were only providing service to people living with HIV, but now we are dealing with almost every health problem”* (CHW, Kibaha).

Having danger sign posters hanging on the walls in the ADDOs was mentioned as a benefit; moreover, dispensers reported that the community has come to recognize ADDOs play a bigger role in helping sick people. Dispensers also felt that they now have closer relationships with pregnant women as opposed to the past when such women were not likely to share their health problems for fear of “bad eye” (a belief that a pregnant woman should keep her pregnancy secret until the baby is born because not everyone wishes her luck in having a child). “*For example, a pregnant woman can come for advice, and I advise her accordingly and teach her some of the danger signs that she should look for. Showing her the posters, she 100 percent believes in my advice*” (dispenser, Kibaha).

#### Seeing more patients

All participants agreed that they now see more patients compared to previous years. They attributed this increase to the community’s improved ability to identify danger signs, likelihood to seek consultations, and the trust they have in their care providers to discuss health needs. For example, one CHW said that “*In the past, not all patients we visited understood and trusted us. You could explain something to the patients, and you see that he is still doubtful, but now we are more confident in explaining health problems, and people follow our referral advice*” (CHW, Kibaha). An ADDO dispenser added, *“Nowadays, I receive many pregnant women with prescriptions for conditions like UTI and vaginal fungus. I think in the past, women used to hide such problems*” (dispenser, Kibaha).

#### Increased sales at ADDOs

As a result of seeing more patients, ADDO dispensers felt that their income has also increased because communities now seek modern treatment as opposed to previously when they preferred traditional medicine: *“At least now you can close the shop in the evening with 30,000 TSh* [about US$15]*. In the past, where the whole day you might end up with 3,000 TSh* [about US$1.50]” (dispenser, Kibaha). In addition, dispensers thought that communities have come to understand the importance of taking the full treatment course as opposed to the past when they often wanted to buy half the amount of medicine called for.

#### More meetings with HF staff and improved services

After the training, CHWs now meet more than once a month with HF staff to report and discuss progress and challenges with different cases they see in the community. As opposed to their previous situation, when they saw only the few patients living with HIV in their village, they now visit pregnant women, newborns, women who have just given birth, children under 5 years, and those in need of family planning services. As a result, CHWs felt proud and viewed themselves as health care professionals.

### Challenges and promising approaches

CHWs and ADDO dispensers said that patients face challenges when they go to the health facility, including lack of medicines. Because of this, ADDO dispensers reported having difficulties advising some patients with danger signs who refused to go to the HF because of the fear that medicines will not be available, “*There was a woman who brought her baby with bleeding umbilical cord, I told her to immediately go to health facility. She was reluctant, emphasizing that I should give her medicines because she will not find any at the health facility*” (dispenser, Kibaha). Other challenges with referrals included CHWs lacking registers to record referred patients, no ambulance services to help patients get immediate help, and weekend referrals to HFs, when adequate staffing can be a problem.

Some ADDO dispensers also felt that CHWs still trusted HF staff members more than them, “*They should also refer patients to us; they tell everyone to go to HF. We are also professional, so they should also send patients to us”* (dispenser, Kibaha). On the other hand, some HF staff had concerns about working with CHWs. One said, *“It is not always easy. You can plan an activity, but when you call the respective CHW to participate, she is not available. Sometimes they are busy with their small business—as you know, they are not paid any salary*” (HF staff member, Kibaha). HF staff also expressed the opinion that working with ADDO dispensers could be difficult because dispensers who were trained in the linkage sometimes left for work in other areas, so they had to cope with new dispensers who were not oriented in the intervention.

While CHWs and ADDO dispensers reported improved relationships after training, the two still had no formal mechanism for discussing progress or challenges with identifying patients and improving timely access to care among mothers, newborns, and children under five. In addition, when asked about supervision to ensure smooth linkages, CHWs and ADDO dispensers reported that they had not received any. HF staff confirmed that they had not done any additional supervision other than the usual monthly meetings with CHWs when presenting their reports. HF staff proposed that supervision occur during staff visits to the respective communities to find out what is happening and also to link CHWs with village offices, so they can receive more support in identifying patients in need. Likewise, although the training established new relationships between ADDO dispensers and HF staff, those groups had not met formally since the training, nor had a forum been established to bring them together.

All participants proposed using mobile phones to help both the supervision process and communication among themselves, such as calling about referred patients. However, instead of jumping first to mobile phones as the approach, some participants expressed the need to sit together with other care providers and discuss the optimal ways for supervision and communication to occur. Participants wanted a platform where the three levels of care providers can meet face-to-face and discuss patients’ challenges and how best to work together. They thought that if the training was aimed at linking the three levels, then they need to frequently meet and update each other; otherwise, the linkage would lose its meaning. One ADDO dispenser said, *“So far we have never met. It is quite crucial for us to meet, we should meet at least once a month. The health facility should initiate this, we are ready”* (dispenser, Kibaha), Similarly, a CHW pointed out, *“Now what was the reason of training us together if we don’t meet? It makes no sense to me….CHWs, dispensers, and health facility staff should regularly meet, as we were told during the training, we are one”* (CHW, Kibaha).

For the sustainability of the CHW–ADDO–HF linkage, we asked participants about their motivation. Interestingly, they all expressed the desire to work together to help reduce disease in their community and improve prompt access to care, especially among women and children. Others said that being a CHW means being the people’s servant because there is no salary, and so they have to have a calling to serve their community. ADDO dispensers also added that apart from making more profits as result of seeing more patients, they also strive to reduce maternal and child mortality, because those who are dying are also their relatives.

### Patients’ experiences with referrals and services provided by CHWs, ADDOs, and health facilities

The majority of focus group participants reported receiving visits from CHWs two or three times a month. Participants acknowledged that the work of CHWs has helped decrease maternal and child health problems and deaths in the community, *“CHWs are well recognized by people; their work is of a huge relevance. Pregnant women for example have come to understand danger signs that needs referral to health facility. Honestly, child mortality has decreased now*” (focus group participant, Kibaha). However, as CHWs reported themselves, women felt that CHWs without bicycles or motorbikes have difficulty visiting all households, especially in pastoral communities, where populations are transient.

With regard to referrals, what women in the focus groups expressed was similar to what CHWs had said, in that a decision to refer to either an ADDO or a health facility is determined by the prevailing health problem. They mostly mentioned pregnant women’s conditions that included bleeding during and after pregnancy, blood pressure, anemia, and stomach ache, while among newborns and children under five, they mentioned malaria, coughing, high fever, and umbilical cord problems. Women indicated that in most cases, CHWs referred to HFs and not to ADDOs. Despite their concerns that they had been provided with oral and not written referrals to the HF, they had been well received by health facility staff—especially when they mentioned the CHW who referred them: “*I think they no longer have referral forms, but we are well received at the health facility, and they congratulate the CHWs on sending us in time before the condition is worse*” (focus group participant, Kibaha). The few women who had been referred to ADDOs by the CHWs also reported being welcomed kindly by dispensers.

Discussion participants expressed their views on the importance of CHWs working together with ADDOs and HFs in improving maternal and child health and family planning services. The women agreed that all three levels of care are important and relevant. One said, *“Yes, the CHWs are following sick people in the communities and sending them to the respective facility. At the health facility, there are different tests. In most cases, health facilities are out of stock of essential medicines, you have to therefore go to the ADDO*” (focus group participant, Kibaha). Lack of medicines at HFs was a challenge, especially when patients are asked to purchase medicines at ADDOs after they had already paid for the yearly community insurance scheme (which can only be used in the health facility).

Focus group members talked about the successes they had experienced related to the linkage intervention, which corresponded to what CHWs, ADDO dispensers, and HF staff had reported; for example, an increase in knowledge of danger signs among pregnant women, newborns, and children under five, which required immediate HF attention. The women indicated that CHWs are now available for all health problems, including family planning, and not just HIV. The group also perceived quicker access to treatment, particularly when CHWs identified cases that needed immediate referral to a HF, resulting in fewer child deaths. Participants also explained that once referred to a HF, treatment is always prompt because one does not need to queue to see the doctor. They reported the same experience when they were referred to ADDOs: “*When you give the prescription to the dispenser, she always leaves everything, especially patients who came to purchase over-the-counter medicines, and she gives you first priority*” (focus group participant, Kibaha).

The mothers in the focus groups proposed a number of interventions to help CHWs, ADDOs, and HFs improve their collaboration that were similar to other study participants’ suggestions, such as providing mobile phones to facilitate communication among the three levels of care; providing CHWs with bicycles or motorbike so they can reach more households; and ensuring that medicines are available at both HFs and ADDOs.

### District health officials’ views of linkage successes and challenges

We interviewed district health officials about how the linkage has been functioning and their supervisory role in ensuring its success. We noted that although officials understood their role in ensuring smooth functioning of the three levels of care, they had not made any supervision visits and therefore were not in a position to understand if the three levels had ever met. When we probed more, they reported a lack of funds for the visits and that they had not included supervision budgets in their yearly district plans. Our findings also showed that district officials had not received any reports from the HFs on referrals received or how the linkage was functioning.

Nevertheless, they thought that the training had brought the three levels of care closer in working together to reduce maternal and child health problems—especially ADDOs and HFs and ADDOs and CHWs, who had not worked together previously, and thought that the linkage should continue. To ensure effectiveness and sustainability of the initiative, they had a number of recommendations: 1) budget for linkage supervision should be included in the next year’s financial plan, 2) CHWs who do not already have them should receive mobile phones to facilitate communication with HFs and ADDOs and to track referred patients, 3) CHWs should have first-aid kits to save the lives of people living far from HFs and ADDOs, 4) special guidelines on how CHMTs should supervise the linkage should be drafted, and 5) ADDOs should be included in village health days to showcase their services.

## Discussion

Our study findings indicate that relationships among the three levels of care improved after the linkage training, especially for ADDO dispensers whose contacts with CHWs and HF staff had been limited and who had been regarded more as business people than as care providers. For example, CHWs and ADDO dispensers started visiting each other to discuss patient case challenges, and CHWs were even guaranteeing the loans of their patients to get medicines from ADDOs. However, while CHWs meet regularly with HF staff, participants noted the lack of mechanism to bring together CHWs, ADDO dispensers, and HF staff to discuss shared interests. This is because the CHMT, which is the entity responsible for facilitating the collaboration, had not organized a forum for them to meet or conducted any supervision visits to ensure a smooth process.

CHWs had various ways to identify patients for referral to either ADDOs or HFs, but the process had some difficulties. Because CHWs work in home-based care for HIV, community members worried about having their HIV status exposed or about being perceived as HIV-positive when they were not, which has also been observed elsewhere in Africa [18]. However, we noted that the community was increasingly recognizing that CHWs also follow people with other health problems related to maternal and child health.

Primary referral to an ADDO is recommended when the HF is far away, so the patient can receive a first dose of lifesaving treatment more quickly [19], and generally, CHWs chose their referrals based on the seriousness of their condition. However, many CHWs preferred to refer patients straight to a health facility no matter what, believing that the patient will get a diagnosis. In addition, most villages in Kibaha district have health facilities located within five kilometers, which helps explain the predominance of CHW referrals to HFs. CHWs’ long-standing relationships with HF staff may have also contributed to that practice.

Documentation of referrals was a challenge among the three levels of care, particularly because of the lack of forms available and the common acceptance of oral referrals. Even if they did not have referral forms, ADDO dispensers were systematically recording referrals to HFs in the patient register book, perhaps because they are required to report their service statistics to the Pharmacy Council.

Our study participants, including the mothers in the focus groups, clearly perceived that changes had resulted from the training that linked the three levels of care. Advantages mentioned most often included improved knowledge of case management, strengthened relationships among the three levels of care, increased numbers of patients seeking care and more timely access to care, and most notably, the belief that the number of deaths of mothers and children had decreased. We did not analyze health facility records to measure trends in maternal, neonatal, and child mortality before and after the intervention, because we would miss deaths that occurred at home, and also because that level of analysis was beyond the scope of our study.

Our findings on linking CHWs and HF are similar to results from a systematic review done by Pallas et al. showing the importance of CHWs working with health facilities to improve prompt access to care in developing countries [10]. Also corresponding to our findings is a study in Tanzania [11] and others in Africa [20–23] that revealed the benefit of community, retail sector, and health facility interventions to improve prompt access to treatment. A review by Smith and colleagues [24] indicated that evidence was limited on which provider-user interventions effectively improved prompt access to malaria treatment; however, our study highlights the importance of bringing together key levels of health care provision, CHWs, health facilities and ADDOs, in improving immediate access to maternal and child care services.

Because of the lack of a control group, the study participants’ perceived successes of our intervention could also be attributed to other programs working on similar activities in the study district; for example, under the Ministry of Health and Social Welfare, Engender Health and community-based distributors were trying to increase access to and use of family planning products; Jpihego was working with health facilities to improve maternal and newborn health; while Save the Children implemented a kangaroo mother care program in the district.

Participants felt that lack of medicines at HFs was the main barrier to the linkage success; in addition, they felt there was a gap when ADDO dispensers trained in the linkage left to work in other regions, and new dispensers were not aware of the CHW-referral program. Participants were also concerned about patients being referred to the HF on weekends and not receiving any treatment, which is a common problem in villages [18]. Other challenges mentioned were lack of ambulance services and the absence of documentation of referrals, which made patient follow-up more difficult.

The primary issue that threatens linkage sustainability is that district health officials, particularly CHMT members whose responsibility it is to facilitate health care linkage, had not followed up on the intervention, so were unaware of how it was running. Findings indicated that although CHMT members recognized the importance of the linkage, they had not prioritized it in their council plans and budget. However, district officials did share some thoughtful recommendations, which if implemented, could strengthen the linkage and contribute to greater reductions in maternal and newborn deaths.

## Conclusions

An integrated approach that brings together CHWs, ADDO dispensers, and HF staff to improve the community’s prompt access to care has led to improvements in stakeholders’ perceptions, relationships, and actions to improve maternal and newborn health. To strengthen this collaboration further, public health campaign platforms should include ADDOs as another community health care provider. In addition, CHMTs need to develop plans for supervision and referral monitoring to increase sustainability of the linkage.

## Abbreviations

ADDO: Accredited drug dispensing outlet
CHMT: Council health management team
CHW: Community health worker
HF: Health facility
